# Investigation of Methodological Factors Potentially Underlying the Apparently Paradoxical Findings on Body Mass Index and All-Cause Mortality

**DOI:** 10.1371/journal.pone.0088641

**Published:** 2014-02-12

**Authors:** Grace Joshy, Rosemary J. Korda, Adrian Bauman, Hidde P. Van Der Ploeg, Tien Chey, Emily Banks

**Affiliations:** 1 National Centre for Epidemiology and Population Health, The Australian National University, Canberra, Australia; 2 Australian Centre for Economic Research on Health, The Australian National University, Canberra, Australia; 3 Sydney School of Public Health, The University of Sydney, Sydney, Australia; 4 Department of Public and Occupational Health, EMGO Institute for Health and Care Research, VU University Medical Centre, Amsterdam, the Netherlands; 5 The Sax Institute, Sydney, Australia; Universität Bochum, Germany

## Abstract

**Introduction:**

Findings regarding the association between overweight and all-cause mortality range from significantly lower to higher risk, compared with body-mass-index (BMI) within the “normal” range.

**Methods:**

We examined empirically potential methodological explanations for these apparently conflicting results using questionnaire and linked mortality data from 246,314 individuals aged ≥45 years in the Australian 45 and Up Study (11,127 deaths; median follow-up 3.9 years). Hazard ratios (HR) for all-cause mortality associated with BMI were modelled according to different methods of accounting for illness at baseline, finer versus broader gradations of BMI and choice of reference group, adjusting for potential confounders.

**Results:**

In analyses using the broad World Health Organization (WHO) categories, the all-cause mortality HR was significantly lower in the overweight category (25.0–29.99 kg/m^2^), than the normal weight (18.5–24.99 kg/m^2^) category. However, in analyses accounting for baseline illness, which excluded those with pre-existing illness at baseline, ever-smokers and the first 2 years of follow up, absolute age-standardised mortality rates varied up to two-fold between finer BMI categories *within* the WHO normal weight category; rates were lowest at 22.5–24.99 kg/m^2^ and mortality HRs increased steadily for BMI above (p_trend_<0.02) and below (p_trend_<0.003) this reference category. Hence, the breadth of the BMI categories used and whether or not baseline illness is accounted for explain the apparent discrepancies between reported BMI-mortality associations.

**Conclusion:**

Using fine BMI categories and the category with the lowest absolute rates as the reference group and accounting for the potential confounding effects of baseline illness is likely to yield the most reliable risk estimates for establishing the independent relationship of BMI to all-cause mortality. These results and those of other studies indicate that a BMI of 22.5–24.99 kg/m^2^, not the broad “overweight” category of 25–29.99 kg/m^2^, was associated with the most favourable mortality risk.

## Introduction

With the distribution of body mass index (BMI) shifting upwards in many populations [1], the epidemic of overweight and obesity is now recognised as one of the most important public health problems that the world faces today. At least half the population of most high-income countries is currently overweight or obese [2] and more than 4% of the burden of disease in these countries is directly attributable to high BMI [3].

The association between obesity, defined as having a BMI of 30 kg/m^2^ or more, and increased risk of death, overall and from heart disease, stroke, and certain cancers is well established [4], [5]. Publications based on broad groupings of BMI and meta-analyses of published data have found significantly lower death rates among people with BMI in the World Health Organization (WHO) category of “overweight” (BMI 25.0–29.9) compared to those with BMI in the broad “normal” range (BMI 18.5–24.9[6]–[8] or 20–24.9[9]). In apparent contrast, large-scale studies of individual participant data, with fine gradations in BMI, have shown that the risk of all-cause mortality is lowest in individuals with a BMI of 22.5–24.9 (i.e. within the normal range) and is significantly higher in those with BMIs both above and below this level [4], [10], [11]. This has led to questions about the ideal BMI for longevity and speculation about the potential protective properties of being overweight or slightly obese [12], [13]. Further, pre-existing disease at baseline and smoking are associated with both decreased BMI and an increased risk of death [14], and studies have varied in the extent to which they have addressed this [15].

This paper uses prospective data from a large cohort of Australian adults aged 45 years and over to explore the extent to which methodological issues may influence quantification of the relationship of BMI to all-cause mortality. It aims to investigate the impact on estimates of absolute and relative risk of: (i) the inclusion or exclusion of individuals with pre-existing disease at baseline and those who ever smoked; (ii) the size of the BMI categories used; (iii) the fineness of age adjustment; and (iv) the degree of adjustment for potential confounding factors. A secondary aim is to provide evidence informing the likely BMI level associated with the lowest all-cause mortality.

## Materials and Methods

The 45 and Up Study is a cohort study of 267,153 men and women aged 45 years and over, randomly sampled from the general population of New South Wales (NSW), Australia. Individuals joined the study by completing a postal questionnaire (distributed from 1 February 2006 to 31 December 2008) and giving informed consent for follow-up through repeated data collection and linkage of their data to population health databases. The study methods are described in detail elsewhere [16]. The 45 and Up Study is fully owned and managed by the Sax Institute. Questionnaires and data access policies are available at https://www.saxinstitute.org.au/our-work/45-up-study/governance/.

Baseline questionnaire data include information on socio-demographic and lifestyle factors, height and body weight, medical and surgical history, functional capacity and physical activity. BMI was calculated from self-reported body weight and height, as weight in kilograms divided by the square of height in metres.

Deaths among study participants were ascertained from the New South Wales Registry of Births, Deaths and Marriages for the period 1 January 2006 to 30 June 2012; the Registry provides data on fact of death and date of death for all deaths in NSW. The mortality data were linked probabilistically to the baseline questionnaire data from the 45 and Up Study by the Centre for Health Record Linkage.

### Statistical methods

Excluding 376 (0.15%) participants with invalid age and/or date of recruitment, data from 266,777 participants from the 45 and Up Study were linked to data on fact of death. Consistent with established methods [4], people with extreme measures of BMI (<15 kg/m^2^ or BMI>50 kg/m^2^) were excluded due to the increased probability of measurement error. After excluding people with missing or invalid BMI (n = 20,441; 7.7%) and confirmed linkage errors (n = 22; 0.01%), data on 246,314 participants were available for the main analyses.

The main outcome was defined as all-cause mortality following recruitment into the 45 and Up Study. Eligible participants contributed person-years from the date of recruitment until date of death or end of follow-up (30 June 2012), whichever was the earliest.

In order to explore the extent to which methodological issues might influence quantification of the relationship of BMI to all-cause mortality, a series of analyses were conducted. To investigate the potential impact on hazard ratio (HR) estimates of baseline illness, leading to both weight loss and increasing mortality (sometimes termed reverse causality [17]), ‘all participant’ analyses were performed with no exclusions; and then ‘healthy participant’ analyses were performed, which excluded those reporting on the baseline questionnaire a doctor-diagnosis of heart disease, stroke, blood clot or cancer other than melanoma and skin cancer, or having ever smoked, and also excluded the first 2 years of follow up. Recommended approaches to reduce bias introduced by smokers and participants with pre-existing illnesses involve restricting the analysis to subjects who have never smoked and excluding deaths that occur during the first several years of follow-up (possibly as a result of conditions that caused lower weights at baseline) respectively [10].

BMI categorisation and the choice of reference category are likely to play important roles in the evaluation of the BMI-mortality relationship [18]. All-cause mortality rates and 95% confidence intervals (95% CI) were calculated for different levels of BMI using two different categorisations of BMI [19]: (i) broad categorisation (WHO weight classification in brackets): 15–18.49 (underweight); 18.5–24.99 (normal weight); 25–29.99 (overweight); 30–50 kg/m^2^ (obese); and (ii) finer categorisation of BMI (WHO weight classification in brackets): 15–18.49 (underweight); 18.5–19.99, 20–22.49 and 22.5–24.99 (normal weight); 25–27.49 and 27.5–29.99 (overweight); 30–34.99 (obese class I); and 35–50 kg/m^2^ (obese class II-III).

Mortality rates for males and females were calculated separately and were age-standardised to the 2006 NSW population, in 5 year age-groups, using the direct method [20].

HRs for all-cause mortality according to BMI at baseline were estimated using Cox regression modelling, in which the underlying time variable was age. For the broad BMI categorisation, we used the WHO normal weight category as the reference group for the HR estimates, consistent with previous publications [6]. For the finer BMI categorisation, we used the lowest age-standardised rate in the healthy participant analyses as the reference group. The HR and 95%CI are shown initially accounting for age, the underlying time variable. A sensitivity analysis examined the type of age adjustment: as a categorical covariate in 5-year groups, as a continuous covariate, or as the underlying time variable. Models are also presented adjusted for additional covariates (where appropriate) including, alcohol consumption [alcoholic drinks/week 0, 1–14, ≥15], annual pre-tax household income [AUD <$20,000, $20,000–$39,999, $40,000–$69,999, ≥$70,000], education [<secondary school graduation, secondary school graduation, trade/apprenticeship/certificate/diploma, university degree or higher], region of residence [major cities, inner regional areas, outer regional/remote areas] and health insurance [private health insurance yes/no]. No adjustment was made for physical activity, high blood pressure or high blood cholesterol as these are among the likely mechanisms in the pathway linking body weight and mortality. Missing values for covariates were included in the models as separate categories, which included alcohol consumption 4,771 (1.9%), annual pre-tax household income 51,553 (4.9% missing; 16% declined to answer), education 3,537 (1.4%), region of residence 55 (0.02%) and health insurance 7 (<0.01%).

The proportionality assumption of Cox regression was verified by plotting the Schoenfeld residuals against the time variable in each model, with a stratified form or time-dependent form of the model used where covariates displayed non-proportionality of hazards. In addition to estimating the category-specific HRs, we estimated the HR associated with each 5 kg/m^2^ increase in BMI for BMIs greater than or equal to the lowest cut-point of the BMI category with the lowest mortality rate, and we also modelled the median BMI for categories as a continuous variable. Tests for trend in the risk of mortality with increasing BMI were done using these models. Martingale residual plots were used to verify the linear functional form of BMI, where BMI was modelled as a continuous variable.

All statistical tests were two sided, using a significance level of 5%. All analyses were carried out using SAS® version 9.3 [21]. Ethical approval for the study was obtained from the NSW Population and Health Services Research Ethics Committee and the Australian National University Human Research Ethics Committee.

## Results

The study population was aged between 45 and 110 years at baseline, with a mean of 63 [SD 11]. The majority of participants (61%) were aged 45–64 years with 10% aged 80 years or older; just under half the cohort (47%) comprised men (Table 1). Nearly two-thirds (62%) were overweight or obese (40% and 22%, respectively), with obesity prevalence substantially lower in those aged ≥ 80 years (12%). The mean BMI was 26.9 [SD 4.9] kg/m^2^. The median follow-up time for the cohort was 3.9 years (mean 4.2 years); a total of 11,127 deaths occurred during 1,042,270 person-years of follow up (1,463 deaths occurred during 248,785 person-years of follow up among healthy participants).

**Table 1:** Characteristics of study population according to body mass index (BMI) category at baseline.

				BMI (kg/m^2^) at baseline					
	15–18.49	18.5–19.99	20–22.49	22.5–24.99	25–27.49	27.5–29.99	30–34.99	35–50	Total
n	3103	6842	30185	53738	55661	41740	39307	15738	246314
Mean age (SD)	67 (14)	63 (13)	63 (12)	63 (12)	63 (11)	62 (10)	62 (10)	60 (9)	63 (11)
Male	25	22	32	45	55	57	49	35	47
Tertiary education	21	27	28	27	25	22	19	16	24
Household income ≥ $70,000	13	21	24	25	26	25	23	20	24
Highest physical activity tertile	35	37	39	37	35	32	27	22	33
≥ 15 alcoholic drinks/week	9	8	10	13	16	17	16	11	14
Residing in major cities	48	51	49	48	45	43	42	39	45
Current smokers	17	11	9	7	6	6	7	7	7
Past smokers	26	27	30	34	37	40	41	40	36
History of cancer	15	12	12	11	11	11	11	11	11
History of CVD	18	12	12	14	15	16	16	17	15

Data are percentage of sample within BMI category unless indicated otherwise.

A history of cardiovascular disease (CVD) at baseline was defined as self-reported heart disease, stroke or blood clot on the baseline questionnaire.

A history of cancer at baseline was defined as self-reported history of cancer other than melanoma and skin cancer on the baseline questionnaire.

**Figure 1:**
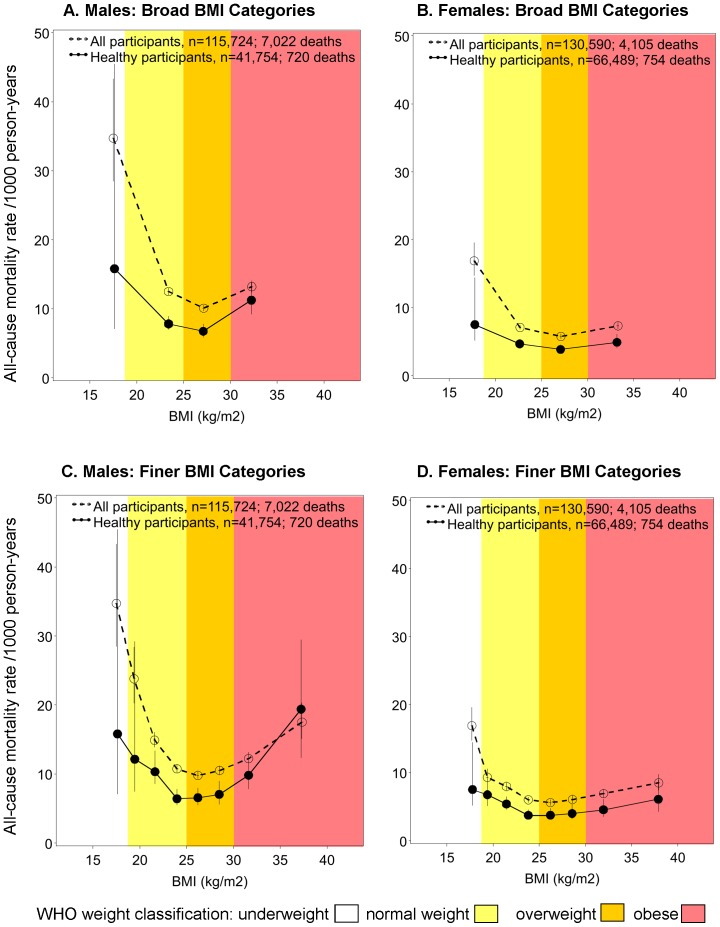
All-cause mortality rates (95% CI) per 1000 person-years by sex and differing BMI categories, directly age-standardised to 2006 New South Wales population. Rates plotted at median body mass index (BMI) for the categories: broad categorisation 15–18.49, 18.5–24.99, 25–29.99, 30–50 kg/m^2^; finer categorisation 15–18.49, 18.5–19.99, 20–22.49, 22.5–24.99, 25–27.49, 27.5–29.99, 30–34.99, 35–50 kg/m^2^. The healthy participants analysis excludes those who ever smoked at baseline, those with a history of CVD at baseline (defined as self-reported heart disease, stroke or blood clot on the baseline questionnaire) and those with a history of cancer at baseline (defined as self-reported history of cancer other than melanoma and skin cancer on the baseline questionnaire), and excludes the first 2 years of follow-up.

The age-standardised rates of all-cause mortality, calculated using the broad WHO categorisation of BMI, were higher in men than in women at all levels of BMI. When all participants were included, the lowest mortality rates were observed in the broad overweight BMI category of 25–29.99 kg/m^2^ for both sexes [deaths per 1000 p-years (95% CI) of 10.1 (9.7–10.5) and 5.8 (5.4–6.1) for men and women respectively; Figure 1a, 1b]. The absolute rates were significantly lower in the healthy participant analysis but the pattern across broad BMI categories remained similar except that there was a greater reduction in the rates among underweight males and females than in the other BMI categories.

**Figure 2:**
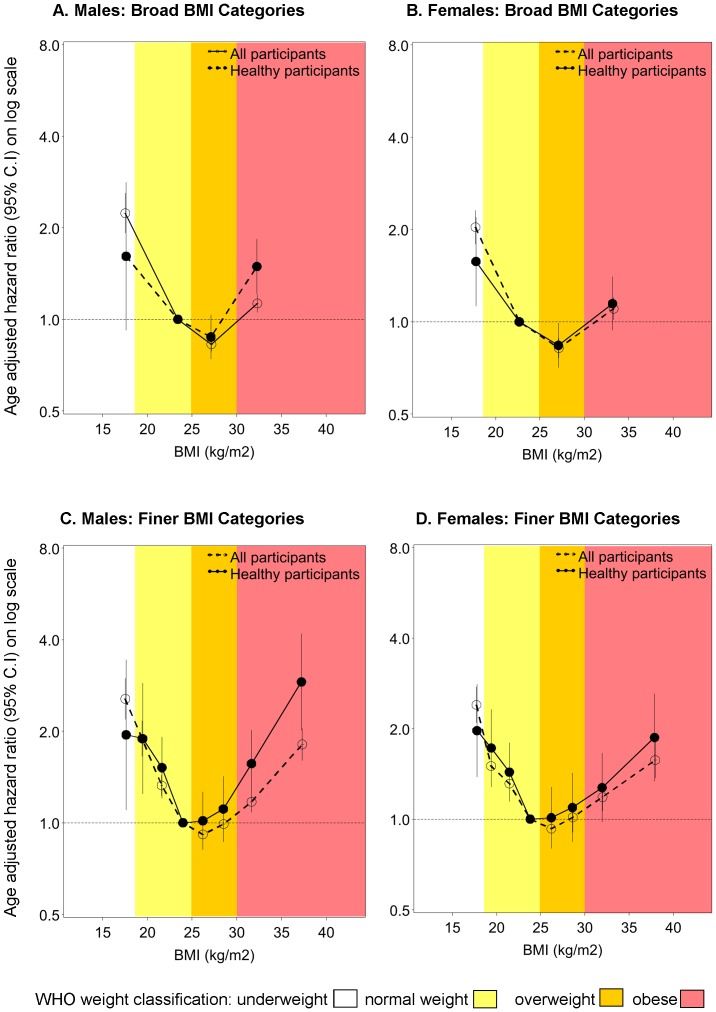
Hazard ratio estimates for all-cause mortality by sex and differing BMI categories. Hazard ratios (HRs), adjusted for age as the underlying time variable, are plotted on log-scale at median body mass index (BMI) for the categories: broad categorisation 15–18.49, 18.5–24.99 (reference), 25–29.99, 30–50 kg/m^2^; finer categorisation 15–18.49, 18.5–19.99, 20–22.49, 22.5–24.99 (reference), 25–27.49, 27.5–29.99, 30–34.99, 35–50 kg/m^2^. 95% CIs are indicated by vertical lines. The healthy participants analyses excludes those who ever smoked at baseline, those with a history of cardiovascular disease at baseline (defined as self-reported heart disease, stroke or blood clot on the baseline questionnaire) and those with a history of cancer at baseline (defined as self-reported history of cancer other than melanoma and skin cancer on the baseline questionnaire), and excludes the first 2 years of follow-up.

In the analyses with broad BMI categorisation, HRs were estimated using the “normal weight” BMI category (18.5–24.99 kg/m^2^) as the reference group. When all participants were included, the age-adjusted HR of all-cause mortality was significantly lower in the overweight category among both sexes compared with the corresponding normal weight category (HR (95% CI): 0.83 (0.79–0.87) and 0.82 (0.76–0.88) standardised for men and women, respectively) and was significantly higher in the obese category (1.13 (1.05–1.21) and 1.10 (1.01–1.20) for men and women, respectively) and in the underweight category (2.23 (1.92–2.60) and 2.03 (1.79–2.31) for men and women, respectively) (Figure 2a, 2b). The pattern remained similar when the data were restricted to healthy participants.

When age-standardised rates and HRs were re-calculated using finer categories of BMI, an up to two-fold variation in the all-cause mortality rates *within* the WHO normal weight category (BMI 18.5–24.99 kg/m^2^) became evident (Figure 1c, 1d, 2c, 2d). When all participants were included in the analysis, the age-standardised rates and HR were lowest in the fine BMI category of 25-27.49 kg/m^2^ [deaths per 1000 p-years (95% CI) of 9.8 (9.3–10.3) and 5.6 (5.2–6.1); HR (95% CI): 0.91 (0.85–0.98) and 0.93 (0.84–1.03) among males and females respectively]. When analyses were restricted to the healthy participants, the lowest age-standardised rates were observed in BMI category 22.5–24.99 kg/m^2^, which was then used as the reference category. The HR (95% CI) of mortality increased steadily above and below the reference category of BMI 22.5–24.99 kg/m^2^, with a HR of 1.46 (1.29–1.65) and 1.23 (1.10–1.37) per 5 kg/m^2^ increase in BMI among males and females respectively [for BMI 22.5 kg/m^2^ upwards, p-trend <0.0001 for males and p-trend = 0.0002 for females (Figure 2c, 2d)].

Compared with models where age is the underlying time variable and is automatically adjusted for, models with age as a continuous covariate or as a categorical covariate resulted in little variation in the HR estimates (Table 2). Adjusting additionally for region of residence, household income, education, alcohol intake and health insurance also resulted in little change in the overall pattern of results, with the greatest change seen in the HRs in the 35–50 kg/m^2^ group (Table 3).

**Table 2:** Sensitivity analysis for coarseness of age adjustment: Hazard ratio estimates (95% CI) for all-cause mortality among healthy participants.

	Male			Female		
BMI	Model 1	Model 2	Model 3	Model 1	Model 2	Model 3
15–18.49	1.94 (1.10– 3.41)	2.06 (1.17– 3.62)	1.95 (1.10– 3.44)	2.04 (1.43– 2.90)	2.02 (1.42– 2.88)	1.97 (1.38– 2.81)
18.5–19.99	1.89 (1.24– 2.88)	1.94 (1.28– 2.96)	1.89 (1.24– 2.88)	1.80 (1.34– 2.42)	1.77 (1.32– 2.38)	1.72 (1.28– 2.32)
20–22.49	1.54 (1.22– 1.94)	1.53 (1.21– 1.93)	1.52 (1.20– 1.92)	1.42 (1.13– 1.78)	1.44 (1.15– 1.80)	1.43 (1.14– 1.80)
22.5–24.99	1.00	1.00	1.00	1.00	1.00	1.00
25–27.49	0.99 (0.80– 1.23)	1.00 (0.81– 1.24)	1.02 (0.82– 1.26)	1.00 (0.79– 1.27)	1.01 (0.80– 1.28)	1.01 (0.80– 1.28)
27.5–29.99	1.10 (0.86– 1.42)	1.09 (0.85– 1.39)	1.11 (0.87– 1.42)	1.08 (0.83– 1.40)	1.08 (0.83– 1.40)	1.09 (0.84– 1.42)
30–34.99	1.52 (1.18– 1.96)	1.51 (1.17– 1.95)	1.56 (1.21– 2.02)	1.22 (0.94– 1.58)	1.24 (0.96– 1.62)	1.27 (0.98– 1.66)
35–50	2.78 (1.93– 4.00)	2.85 (1.98– 4.10)	2.91 (2.02– 4.19)	1.81 (1.29– 2.53)	1.86 (1.33– 2.60)	1.87 (1.34– 2.62)

Excludes those who ever smoked at baseline, those with a history of cardiovascular disease at baseline (defined as self-reported heart disease, stroke or blood clot on the baseline questionnaire), those with a history of cancer at baseline (defined as self-reported history of cancer other than melanoma and skin cancer on the baseline questionnaire) and the first two years of follow up.

Model 1: Follow up time is the underlying time variable, age adjusted for as categorical variable in 5-year groups.

Model 2: Follow up time is the underlying time variable, age adjusted for as continuous variable.

Model 3: Age adjusted for as the underlying time variable.

**Table 3:** Sensitivity analysis for additional covariate adjustment: Hazard ratio estimates (95% CI) for all-cause mortality among healthy participants.

	Male				Female			
BMI	Deaths	Crude rate	HR^1^	HR^2^	Deaths	Crude rate	HR^1^	HR^2^
15–18.49	13	30.16	1.95 (1.10–3.44)	1.85 (1.04– 3.27)	39	15.83	1.97 (1.38–2.81)	1.93 (1.35– 2.76)
18.5–19.99	25	20.57	1.89 (1.24–2.88)	1.84 (1.20– 2.81)	62	9.79	1.72 (1.28–2.32)	1.74 (1.29– 2.35)
20–22.49	122	14.63	1.52 (1.20–1.92)	1.49 (1.18– 1.88)	152	5.99	1.43 (1.14–1.80)	1.45 (1.16– 1.82)
22.5–24.99	171	7.67	1.00	1.00	150	4.23	1.00	1.00
25–27.49	157	5.85	1.02 (0.82–1.26)	0.99 (0.80– 1.23)	129	4.34	1.01 (0.80–1.28)	1.01 (0.80– 1.28)
27.5–29.99	101	5.26	1.11 (0.87–1.42)	1.08 (0.84– 1.38)	87	4.22	1.09 (0.84–1.43)	1.06 (0.81– 1.38)
30–34.99	95	6.78	1.56 (1.21–2.02)	1.45 (1.12– 1.88)	90	4.06	1.27 (0.98–1.66)	1.21 (0.93– 1.57)
35–50	36	9.30	2.91 (2.02–4.19)	2.61 (1.81– 3.78)	45	4.26	1.87 (1.34–2.62)	1.66 (1.18– 2.33)
5 unit increase from			1.46	1.41			1.23	1.17
BMI 22.5 kg/m^2^			(1.29–1.65)	(1.25–1.60)			(1.10–1.37)	(1.05–1.31)

Crude rates are per 1000 person-years.

HR^1^: Hazard ratio (HR) adjusted for age (underlying time variable).

HR^2^: Hazard ratio (HR) adjusted for age (underlying time variable), region of residence, household income, education, alcohol intake and health insurance.

Excludes those who ever smoked at baseline, those with a history of cardiovascular disease at baseline (defined as self-reported heart disease, stroke or blood clot on the baseline questionnaire) and those with a history of cancer at baseline (defined as self-reported history of cancer other than melanoma and skin cancer on the baseline questionnaire) and the first two years of follow up.

## Discussion

In this large prospective population-based cohort study, substantial variation in the relationship of BMI to all-cause mortality was observed according to whether or not individuals with pre-existing disease at baseline were included in the analysis, the coarseness of BMI categories used and the reference BMI category chosen.

Using broad BMI categorisation among all participants and among healthier participants, the lowest mortality rates were observed in the WHO overweight BMI category of 25–29.99 kg/m^2^. However, when age-standardszed rates and HRs were re-calculated using finer categories of BMI, an up to two-fold variation was observed *within* the WHO normal weight category.

With finer BMI categories, the category at which the rates and HR were lowest varied according to whether or not individuals with pre-existing disease at baseline were included in the analysis (25–27.49 kg/m^2^ when all participants were included; 22.5–24.99 kg/m^2^ when individuals with pre-existing disease at baseline, ever-smokers and the first 2 years of follow-up were excluded). When individuals with pre-existing disease at baseline, ever-smokers and the first two years of follow up were excluded from analyses and finer BMI categories were used, there was at least a 25% linear increase in the risk of all-cause mortality for every 5 unit increase in BMI, from BMI 22.5 kg/m^2^ onwards. The coarseness of age adjustment or adjusting additionally for other potential confounders, including region of residence, household income, education, alcohol intake and health insurance, did not materially modify the results.

We are not aware of any previous large-scale studies that have investigated empirically the factors underlying the apparently conflicting results on the association between BMI and mortality. A study based on 11,000 people from the National Population Health Survey of Canada examined findings for both broad and finer categorisation of BMI, included covariate adjustment for smoking status and performed sensitivity analyses excluding the first four years of follow-up to account for baseline illness. However, power was limited, particularly for the finer categories of BMI, and the analysis pertaining to finer categorisation of BMI did not account for baseline illness [8]. Nevertheless, our findings relating to the relationship of BMI for broad [6], [8], [22] and fine categorisations of BMI [4], [11], [23], [24] generally agree with the published evidence for each of these categorisation types, respectively. Similarly, our findings for analyses that do and do not take account of baseline illness and smoking status are also consistent with published studies [4], [6], [11], [23], [24].

Particularly worth noting is the recent meta-analyses of published data, using broad categories of BMI [6] and recent pooled analyses of individual participant data, using fine BMI categories [4] [11]. The meta-analysis using broad WHO BMI categories [6] based on 2.88 million participants and >270 000 deaths reported that, compared with normal BMI, overweight was associated with significantly lower all-cause mortality [HR (95% CI) 0.94 (0.91–0.96)], class one obesity (BMI 30.0–34.9) did not differ significantly [0.95 (0.88–1.01)] and classes 2 and 3 obesity (BMI ≥35) were associated with significantly increased mortality [1.29 (1.18–1.41)]. In this meta-analysis, where individual data were not available, adjustments for confounding factors were possible only by excluding whole studies. The authors stated that the results did not change materially when smokers and those with a history of cancer or heart disease were excluded [5].

In both the pooled analyses based on fine BMI categorisation [4], [11], the lowest death rates were observed for BMI in the normal weight range. Since individual data were available, these analyses allowed covariate adjustments and restrictions. The Prospective Studies Collaboration combined individual data on 900,000 adults from 57 prospective studies, adjusting for smoking and excluding person-years and deaths in the first five years from the main analyses, to limit the effect of baseline illnesses [4]. They concluded that all-cause mortality increased with increasing fine categories of BMI beyond a BMI of 22.5 kg/m^2^ and that the lowest death rates were observed for BMI 22.5 to 24.9 kg/m^2^ in both sexes. They noted the potential for the BMI with the lowest underlying death rates to be even lower than this, if the influence of weight loss secondary to disease were to be eliminated completely. The other analysis based on pooled individual data from 19 prospective studies, which included 1.46 million white adults, restricted their data to participants who never smoked and were not diagnosed with cancer or heart disease [11]. It also concluded that both overweight and obesity were associated with increased all-cause mortality, with lowest mortality in the BMI range of 20.0 to 24.9 kg/m^2^. Hence, although the methods of dealing with baseline illness differed between the two pooled analyses, their findings were consistent.

Based on our study as well as previous literature, the central questions relevant to quantifying the ideal BMI for longevity include: (i) how should BMI be categorised? and (ii) should baseline illness be accounted for?

First, let us consider the statistical implications of categorising a continuous variable in regression analyses. When categorising a continuous variable, one assumes that the relation between the independent and the dependent variable is constant within each interval, since any change in effect *within* an interval will not be quantified [25], even a biologically plausible one. Statistical efficiency increases with the number of categorical groups [26], [27] and is usually greatest using a continuous analysis (assuming that assumptions such as linearity are satisfied). More practical considerations, however, may favour categorisation for ease of interpretation and may be motivated by use of clinically relevant cut-off points [28]. In the case of the previously mentioned meta-analysis of published data, categorisation according to WHO groups was necessary since these were the categories used in the original publications [6].

Are categories based on WHO BMI cut-offs a good choice when looking at the association between BMI and mortality? In 1999, the Expert Committee of WHO [29] proposed a BMI classification with cut-offs of 25, 30, 35 and 40 kg/m^2^ in adults for Pre-obesity, Obesity classes I, II, and III respectively, based principally on initial analyses of the association between BMI and mortality. The main basis of the WHO BMI classification [29] were two papers: the first paper was a 1997 publication by Gill [30], which included a figure adapted from the Nurses’ Health study [10], demonstrating that when biases are removed from analysis, an almost linear, continuous relationship between BMI and mortality is found, with no specific lower threshold; the second was a review paper by the American Institute of Nutrition, which concluded that the lowest mortality is seen at BMI 18–25 kg/m^2^ [31]. In the Nurses’ Health study, (based on women aged 30–55 years, followed up for 16 years from 1976), BMI<19 was used as the reference group for relative risk estimation. Bias from reverse causality was limited by restricting analyses to: women who never smoked, with weight change of not more than 4 kg from 1976–1980, who were free of known cardiovascular disease and cancer, and by excluding the first four years of follow-up.

Estimating the regression coefficients for levels of a categorical predicator involves specification of an appropriate reference category against which the other categories are compared [32], especially for nominal categorical predictors [33]. Both the biological interpretation of the estimated association and the number of observations should be considered when choosing the reference category [25]. Although the Expert Committee of WHO defined 18.50–24.99 kg/m^2^ as the normal range for BMI, it was also noted that the broad ranges of BMI do not imply that an individual can fluctuate within this range without consequence; for example, for an individual 1.75 m tall, the BMI range of 18.50–24.99 covers a weight range of 20 kg. The Expert Committee of WHO had also identified BMI standards for the elderly (>60 or >80 years) as a priority area for future research. The observed variations within the WHO normal weight category BMI 18.5–24.99 kg/m^2^ indicate that it is too broad to be a suitable reference category in the analysis of BMI and mortality, especially for studies that include older people aged 60+ years. Modelling BMI as a continuous variable employing regression techniques that use restricted cubic splines is likely to be of interest for future analyses, but is beyond the scope of the current paper, which seeks to explain the differences between published findings on BMI and mortality [34].

People frequently lose weight as a result of an illness that is ultimately fatal, a situation that creates the appearance of higher mortality among those with lower weights [14] and the leanest group in a population is a mix of smokers, people who have lost weight as a result of underlying disease, and people who have maintained a lean weight by balancing physical activity and caloric intake [14]. Because both BMI and mortality are likely to vary in a graded manner with the severity of pre-existing illness, simple binary adjustment for the presence or absence of disease will not adequately deal with such confounding. It follows that people who have lost weight due to illness are likely to be distributed across the BMI spectrum and it is difficult, if not impossible, to estimate the independent effects on mortality of high BMI in people who are ill.

In prospective studies, the best analytic approach depends on what is already known across a variety of disciplines about the relationship under examination and the statistical implications of analytical choices. Inadequate control for pre-existing illness and smoking status can distort the true relation between body weight and the risk of death because chronic illness and smoking are associated with both decreased BMI and an increased risk of death [14]. Suggested strategies [14] for prospective studies to address the issue of bias due to pre-existing baseline illness are: excluding participants with diagnoses that might affect weight; excluding participants who report recent weight loss (such as during the previous five years) [10]; and excluding deaths that occur during the first several years of follow-up (possibly as a result of conditions that caused lower weights at baseline) [10]. Restricting the analysis to participants who have never smoked [10] or reporting the results in subgroups of smoking status are other strategies. Concerns regarding accounting for pre-existing illness at baseline have been raised citing weak evidence of occurrence of bias, disproportionate exclusion of overweight/obese participants, insufficient evidence regarding the adjustment strategies about their validity and systematic effects on relative risks [35]. However, evidence based on simulation studies [17] and large prospective studies [24], [36] have demonstrated that bias is introduced by inclusion of participants with pre-existing illnesses and it needs to be accounted for. Furthermore, this study has demonstrated that excluding people with baseline illness results in substantive effects on the estimates of the BMI-mortality relationship with fine BMI categorisation, but not with broad BMI categorisation. If there really was no bias due to reverse causality or confounding by baseline illness, one would expect comparable results in people with and without baseline illness and consistent results regardless of exclusion of people with baseline illness. The WHO classification of BMI [29] is also based on a study [10] which accounted for pre-existing illness at baseline and smoking.

Use of the standard broad BMI categories in the analysis of BMI and mortality may appear to facilitate between-study comparisons and make decision making in a clinical setting simpler. However, a major drawback of the use of broad BMI categorisation is that the statistical assumption of a constant relationship within intervals in the categories of continuous variables is violated. Large variations in absolute rates and two-fold variation in the HR of death were observed within the broad normal BMI category (which the WHO had cautioned about), indicating that the relation between mortality and BMI is not constant within the normal BMI category.

It should be borne in mind that the WHO BMI categories were originally defined based on evidence from an observational study and the WHO group themselves noted potential for revision of the cut-offs, should evidence indicative of a need for change emerge [29]. Indeed, the categories have been revised downwards for Asian populations [37].

Analytic strategies to minimise bias due to pre-existing illness at baseline, use of finer BMI categories and analysis restricted to participants who never smoked are likely to yield estimates of BMI and mortality relationship that are more reliable for estimating the likely independent effects of BMI in the population. Ideally the reference group for finer categories should be the group with the lowest absolute mortality rate among healthy participants; however, studies which include people aged over 60 years require careful consideration due to possible high prevalence of pre-existing illnesses at baseline.

Finally, a model with time-on-study, adjusting for age as a continuous variable, assumes that age has a log-linear association with the hazard at study entry. It also assumes that the effect of follow-up time is similar across the different ages (e.g. the proportional increase in the hazard over one year is the same for someone aged 45 years and 85 years). Further, variability in the age-at-entry of individuals in the study could cause the models to differ significantly [38], even when age is adjusted for [39]. These assumptions and problems can be avoided with the use of age as the underlying time scale. Even though it is computationally intensive in practice, using age as the time scale gives more accurate results because it puts similar subjects in the risk set together and allows a completely non-parametric age effect [40]. Alternatively, age could be modelled as a non-linear predictor, employing regression techniques that use restricted cubic splines. When analysing studies with longer follow up times, it should also be noted that none of the above mentioned approaches take cohort effects into account and the potential for the BMI-mortality relationship to vary across birth cohorts cannot be excluded.

Major strengths of this study are: (i) its large sample size (which means that we were able to make the appropriate exclusions and stratification), (ii) the availability of a range of potential confounders measured at baseline, allowing covariate adjustments, and (iii) virtually complete follow up data on death. However, due to the relatively short follow up period at the time of analysis, we were only able to exclude the first two years of follow up to control for sources of bias arising from pre-existing disease at baseline. BMI was calculated using self-reported weight and height and data on potential confounding factors were mostly based on self-report. Regarding self-reported BMI, although people tend to underestimate their weight and overestimate their height [41], and consequently underestimate BMI, a validation study involving participants in the 45 and Up Study revealed that the mean difference between self-reported and measured BMI was not large (on average –0.74 kg/m^2^) and correlations between self-reported and measured height and weight were 0.95 and 0.99, respectively [42]; results which are consistent with other studies. Other measures of adiposity, such as those relating to central obesity, were not ascertained in our study. The independent effect of BMI may be slightly overestimated as factors such as physical activity will also exert an independent effect on mortality. The central purpose of this paper is not the definitive quantification of the risk of mortality according to BMI, but to demonstrate the potential differences in results depending on methodological choices. It is important to refer to studies with appropriate statistical power and accounting for BMI measurement error, for example [4], for statistically reliable evidence on the BMI-mortality relationship.

In conclusion, evidence from this study indicates that investigations of the relationship between BMI and mortality could easily arrive at opposing conclusions on the mortality risk of being overweight but not obese, depending on the coarseness of BMI categories used and the measures implemented to control bias. We have demonstrated that study exclusions to minimise bias (pre-existing illness at baseline and smoking), coarseness of BMI categorisation and choice of reference category had a significant impact on the relative hazard estimates. Paradoxical associations suggesting a beneficial association of overweight with mortality were observed in models where sources of bias were inadequately controlled and broad categories of BMI were used. If BMI is modelled as a categorical variable in the analyses of mortality risks in relation to BMI, the use of fine classification of BMI is recommended, along with strategies to minimise bias due to pre-existing illness and factors such as smoking.
